# Host-Dependent Medicinal Quality of Taxilli Herba: A Comprehensive Review on Chemical Profiles and Bioactivities

**DOI:** 10.3390/biom16081131

**Published:** 2026-08-03

**Authors:** Mei Ru, Rong Su, Min Guo, Zhihui Yin, Wendong Li, Ping Li, Zishu Chai, Yonghua Li

**Affiliations:** 1 College of Pharmacy, Guangxi University of Chinese Medicine, Nanning 530200, China; rum@gxtcmu.edu.cn (M.R.); surong2024@stu.gxtcmu.edu.cn (R.S.); guom@gxtcmu.edu.cn (M.G.); yinzhihui2024@stu.gxtcmu.edu.cn (Z.Y.); liwendong2025@stu.gxtcmu.edu.cn (W.L.); liping2024@stu.gxtcmu.edu.cn (P.L.); 2 Cenxi Sangjisheng Agri-Tech Courtyard, Cenxi 543200, China; 3 Guangxi Key Laboratory of Efficacy Study on Chinese Materia Medica, Guangxi University of Chinese Medicine, Nanning 530200, China; 4 Faculty of Chinese Medicine Science, Guangxi University of Chinese Medicine, Nanning 530200, China

**Keywords:** host trees, materia medica, medicinal material quality, quality management, Taxilli Herba

## Abstract

Taxilli Herba (Sangjisheng) is a hemi-parasitic medicinal plant whose quality is intrinsically linked to its host species. While previous reviews have extensively summarized its chemical constituents and pharmacological activities, a systematic understanding of how host trees dictate its quality and safety remains largely fragmented. This review bridges this gap by proposing a “host-governed quality paradigm.” By integrating historical records with modern scientific findings, we critically evaluate the molecular mechanisms underlying host–parasite interactions and their impact on medicinal quality. We highlight that host species influence Taxilli Herba across three critical dimensions: (i) chemical composition, comprising both intrinsic bioactive compounds and host-derived metabolites; (ii) toxicity profile, where even hosts lacking cardiac glycosides may pose potential safety hazards; and (iii) pharmacological activity, where host identity modulates therapeutic potency and traditional medicinal properties (e.g., “cold” vs. “warm” nature). Importantly, we highlight that the cardiac glycoside test specified in the current Chinese Pharmacopoeia is insufficient to ensure the safety of Sangjisheng derived from diverse hosts. To address these challenges, we propose a host-governed quality paradigm comprising four pillars: (1) mechanistic studies on host–parasite interactions; (2) revision of pharmacopeial standards to restrict approved sources; (3) promotion of standardized cultivation using safe host species (e.g., *Morus alba*); and (4) stringent quality control for edible and medicinal applications. This framework aims to ensure the safety, efficacy, and sustainable use of Sangjisheng in clinical practice.

## 1. Introduction

In the extensive domain of traditional Chinese medicinal herbs, Taxilli Herba, known as Sangjisheng, holds a pivotal role. This herb is derived from the dried leafy stems and branches of the *Taxillus chinensis* (DC.) Danser, which belongs to the Loranthaceae family [1]. Its medicinal usage has a rich history, tracing back over 2000 years to the Shennong Bencao Jing of the Eastern Han Dynasty [2]. Sangjisheng is renowned for its diverse therapeutic properties, including dispelling wind-dampness, tonifying the liver and kidneys, strengthening tendons and bones, and calming the fetus [1,3]. In clinical practice, it is widely utilized for various conditions, such as rheumatic pain, weakness of the lower back and knees, tendon and bone debility, excessive menstrual bleeding, threatened abortion, fetal irritability, dizziness, and blurred vision [1]. It serves as a vital ingredient in numerous traditional Chinese medicine formulations, providing relief to countless patients.

**Figure 1:**
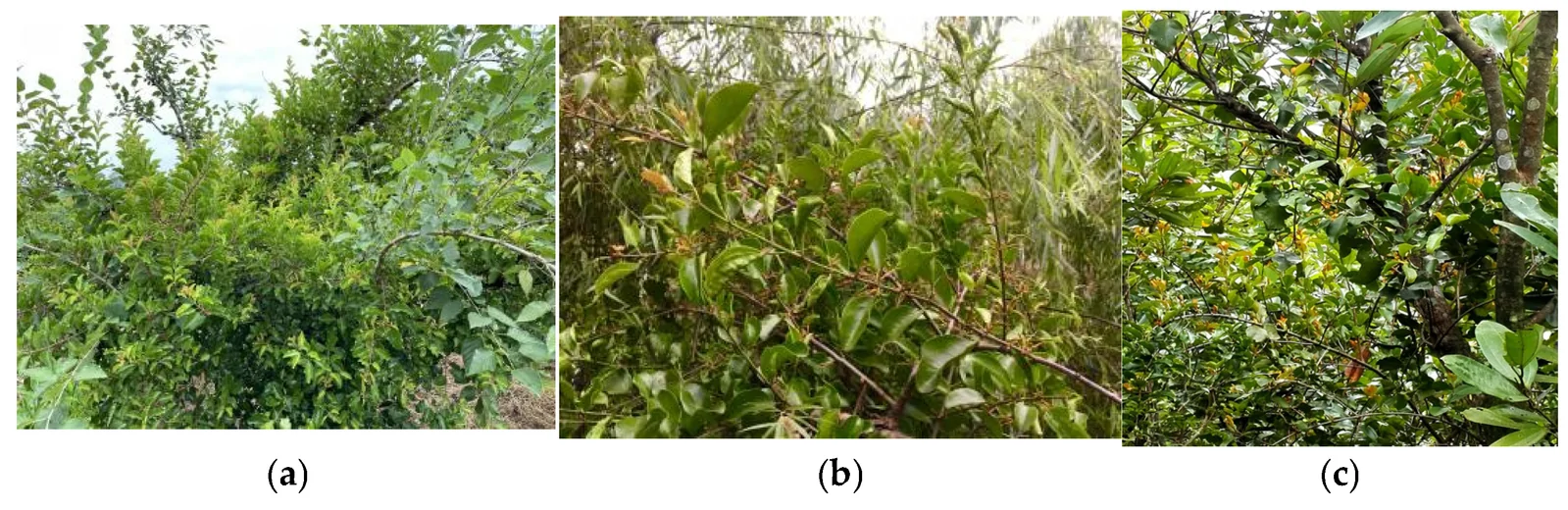
Sangjisheng parasitizing mulberry (**a**), willow (**b**), and cinnamon (**c**) hosts.

As a hemi-parasitic medicinal plant, *T. chinensis* exhibits a complex and diverse range of host trees [4]. In the natural habitats of Guangxi, China, the host trees of *T. chinensis* encompass over 35 families and more than 150 species, including mulberry (*Morus alba*), willow (*Salix* spp.), cinnamon (*Cinnamomum cassia*), peach (*Prunus persica*), plum (*Prunus mume*), longan (Dimocarpus longan), and litchi (*Litchi chinensis*) [4,5] (Figure 1). The influence of host plant species on the quality of Sangjisheng has consistently been a primary concern for medical practitioners [4]. Although Sangjisheng is inherently non-toxic, it may accumulate toxic constituents when parasitizing toxic hosts, thereby posing a risk to medication safety [6]. Ensuring the quality and safety of Sangjisheng, particularly when given its use in preventing threatened abortion, has emerged as a critical issue in quality management [7]. Consequently, it is imperative to investigate the effects of host species on the quality of Sangjisheng and to propose effective quality management strategies.

However, despite the growing body of research on this herb, a systematic understanding of how host trees dictate its quality and safety remains largely fragmented. While previous reviews have extensively summarized the chemical constituents and pharmacological activities of Taxilli Herba, they have not fully addressed the precise knowledge gap regarding the lack of a unified framework explaining host-dependent variation in metabolites and toxicity [8]. To bridge this gap, this review adds value by proposing a “host-governed quality paradigm.” Crucially, we distinguish between historical records (empirical evidence from traditional materia medica) and modern scientific evidence (metabolomics and in vivo toxicological assessments). Unlike traditional overviews, we critically evaluate the molecular mechanisms underlying host–parasite interactions and provide actionable regulatory recommendations based on host-specific quality markers.

## 2. Materials and Methods

### 2.1. Literature Search Strategy

To comprehensively capture the historical evolution and modern scientific evidence regarding Taxilli Herba, the literature search was conducted in two parts:

(1) Modern Scientific Literature

A systematic search was performed to identify relevant studies published between 1980 and April 2026. The following electronic databases were searched: PubMed, Web of Science, Scopus, and CNKI. The search strategy combined keywords and MeSH terms (or TCM terms for CNKI) using the following algorithm: (“Taxilli Herba” OR “Sangjisheng” OR “*Taxillus chinensis*”) AND (“host tree” OR “parasitic plant”) AND (“quality” OR “metabolites” OR “toxicity”).

(2) Historical TCM Classics

To trace the historical origin of the “host-governed quality paradigm,” the classical Chinese herbal literature (e.g., Shennong Bencao Jing, Bencao Gangmu) and historical medical texts were manually reviewed. These ancient texts were identified through specialized TCM databases (e.g., Chinese Medical Classics Database) and traditional bibliographic references.

Inclusion criteria were as follows: (1) studies focusing on the relationship between host trees and the quality/toxicity of Taxilli Herba; (2) original research articles, reviews, or meta-analyses; (3) articles published in English or Chinese. Exclusion criteria were: (1) duplicate publications; (2) studies lacking specific data on host plants or chemical composition; (3) conference abstracts, editorials, and case reports.

The titles, abstracts, and full texts were independently screened by Mei Ru and Rong Su to ensure the relevance and quality of the included studies. Any discrepancies were resolved through discussion or consultation with a third reviewer. To systematically evaluate the reliability and relevance of the collected literature, we categorized the evidence into distinct types, ranging from historical records to modern clinical trials. Table 1 outlines these evidence types, detailing their respective methodologies, contributions to the “host-governed quality paradigm,” and inherent limitations. This classification allows for a more rigorous integration of traditional wisdom with modern scientific validation.

### 2.2. Use of AI and Software Tools

**Figure 2:**
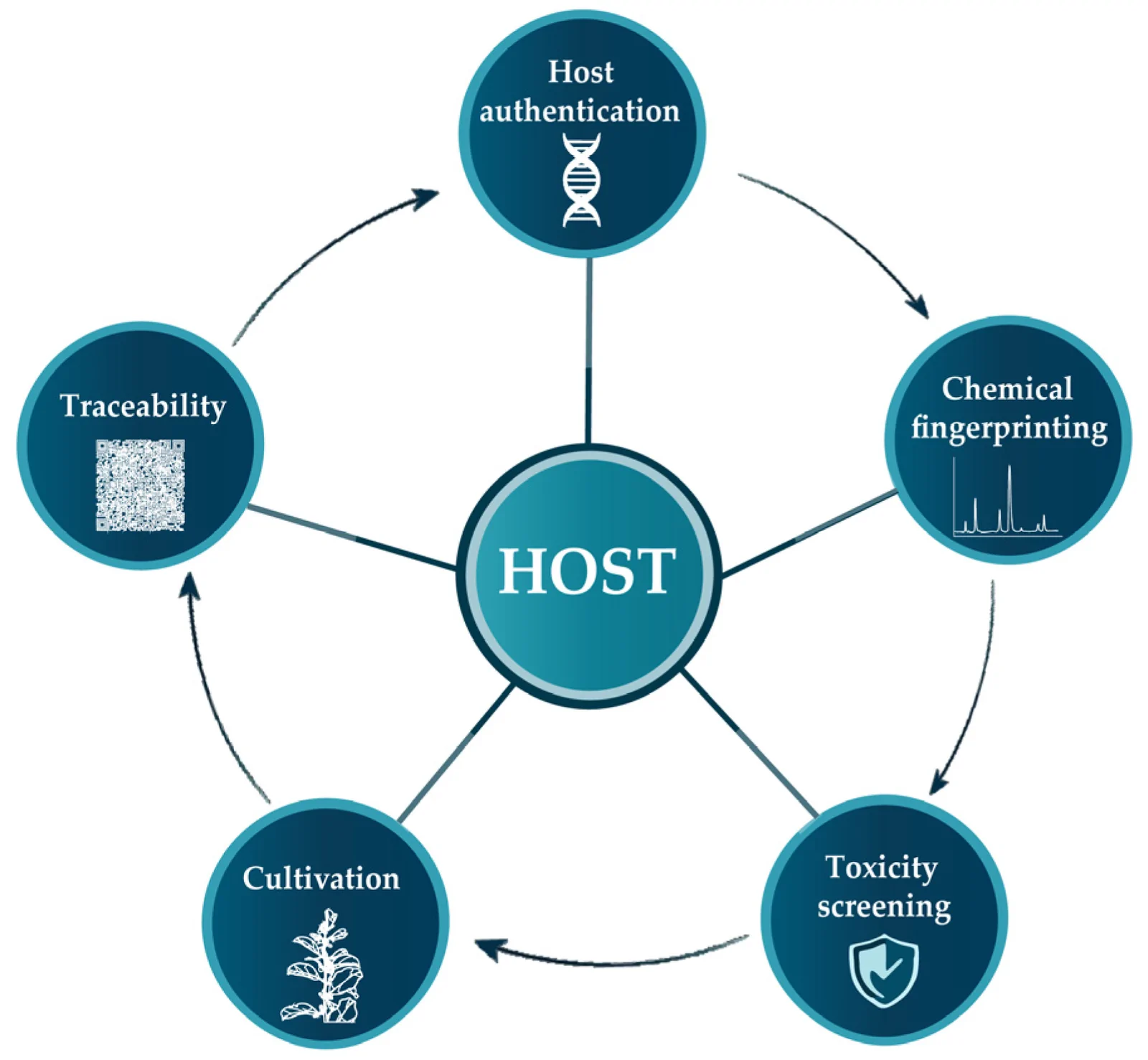
Schematic diagram of the host-governed quality paradigm for Sangjisheng. This framework integrates five key modules—Host authentication, Chemical fingerprinting, Toxicity screening, Cultivation, and Traceability—to establish a systematic approach for quality control. By centering on the host plant, this paradigm addresses both intrinsic toxicity and extrinsic safety risks (e.g., environmental contaminants), ensuring the safety and consistency of Sangjisheng products. Note: the cyclic arrows indicate the interactive and iterative relationship among all research modules. The QR code icon serves only as a schematic representation of traceability management.

During the preparation of this work, the authors utilized Qwen3.7 for the purposes of grammar checking, spelling and punctuation correction, and language optimization. Additionally, Qwen3.7 was also employed to assist in brainstorming the logical framework and schematic layout for the Graphical Abstract and Figure 2. However, the final visual design, illustration, and assembly of the figures were manually executed by the authors using Adobe Illustrator 2026.

## 3. Historical Overview of Sangjisheng in Materia Medica

### 3.1. Nomenclature and Historical Context

The medicinal history of Sangjisheng can be traced back to the Shennong Bencao Jing of the Eastern Han Dynasty, where it was referred to as “Sangshang Jisheng”, explicitly indicating its parasitic growth on mulberry trees in river valleys [2]. Subsequently, the Mingyi Bielu recorded “Sangshu Jisheng”, noting its growth on mulberry trees in Hongnong, which further emphasizes its specific habitat [9]. The Bencao Jing Jizhu stated, “Those growing on mulberry trees are called Sangshang Jisheng.” It also noted that practitioners utilized specimens from poplar and maple trees, naming them according to their host tree, as their sap and properties vary depending on the location [10]. This indicates that ancient scholars not only recognized the ability of Sangjisheng to parasitize various trees but also classified and named it based on its host. The Bencao Gangmu (Compendium of Materia Medica) documented Sangjisheng, Liujisheng, and Taojisheng, parasitizing mulberry, willow, and peach trees, respectively [11]. This practice of naming Sangjisheng medicinal materials according to their host reflects the rigorous distinction made by ancient physicians regarding the source of Sangjisheng, as they believed that only that parasitic on mulberry trees constituted authentic medicinal material. This understanding has provided a crucial basis for subsequent generations to identify Sangjisheng. Critically, this historical emphasis on the mulberry host was not merely a botanical classification, but an early empirical recognition of host-dependent pharmacological variations. As modern phytochemical research will demonstrate, this ancient preference for mulberry aligns with its unique accumulation of specific bioactive metabolites [12], providing a scientific basis for traditional authenticity.

### 3.2. Historical Perspectives on Toxicity

Historical herbal texts have meticulously documented the potential toxicity of Sangjisheng depending on its host tree. The Bencao Gangmu states that while Sangjisheng growing on mulberry trees is usable, substitutes from other trees possess different properties and “may, on the contrary, be harmful” [11]. This explicitly indicates that Sangjisheng parasitic on mulberry trees is non-toxic, whereas that from miscellaneous trees may pose health risks. The Zhenglei Bencao (Classified Materia Medica) provides a starker warning through a specific case study. It notes that although parasitic plants grow on oak, willow, poplar, and maple, only the variety from mulberry trees is suitable for medical use. The text recounts a tragic incident where a counterfeit substitute from another tree was deceptively provided to a patient, resulting in the recipient’s death within a month of consumption [13]. This serves as a grim reminder of the dangers of misidentified hosts. From the Shennong Bencao Jing to the Classified Materia Medica, the consensus remains that true Sangjisheng (on mulberry) is non-toxic, whereas that from other hosts must be approached with extreme caution, underscoring the critical link between the host species and toxicity. This historical consensus on host-specific toxicity has been strikingly validated by modern toxicological studies. Contemporary analyses reveal that while mulberry-derived Sangjisheng is pharmacologically safe, samples from non-mulberry hosts (such as willow or oleander) often contain distinct, potentially lethal secondary metabolites (e.g., cardiac glycosides). Thus, the ancient warnings against misidentified hosts serve as a profound pre-modern warning of what we now define as host-induced hepatotoxicity and cardiotoxicity [14,15].

### 3.3. Variation in Therapeutic Efficacy

Ancient texts provide detailed descriptions of the varying efficacies of Sangjisheng parasitic on different hosts. The Bencao Jing Jizhu vividly illustrates that parasitic medicinal materials derived from different host tree species exhibit therapeutic properties, as captured by the phrase “root sap varies according to location.” [10]. The Bencao Gangmu documents three distinct varieties of Sangjisheng, each exhibiting unique indications. Sangjisheng parasitic on mulberry trees is primarily indicated for “treating lumbago, rigidity in children’s backs, abscesses, nourishing the skin, strengthening hair and teeth, promoting beard and eyebrow growth, and stabilizing pregnancy.” In contrast, the variety on willow trees is mainly used for “alleviating diaphragmatic qi stagnation and stabbing pain,” while that on peach trees is primarily indicated for “treating gu poison in children, abdominal hardness and pain, a greenish-yellow complexion, and emaciation.” [11]. The Diannan Bencao further elaborates on these host-dependent differences. For instance, the parasite on mulberry trees is described as “treating bone and muscle pain, promoting meridian circulation, and alleviating wind-cold-dampness bi syndrome (arthralgia).” Conversely, the variety on Chinese Scholar Trees (*Styphnolobium japonicum*) is noted for “primarily treating lower intestinal bleeding, hematochezia due to intestinal wind, and hemorrhoids and fistulas.” Furthermore, the parasite on Chinese Prickly Ash (*Zanthoxylum bungeanum*) is characterized by its ability to “treat cold deficiency of the spleen and stomach, vomiting, nausea, and regurgitation.” [16]. These records collectively demonstrate that ancient practitioners possessed a profound understanding of how host species determine the therapeutic profile of parasitic plants. While the historical preference for mulberry-hosted Sangjisheng is well-documented, the question remains: does modern evidence support this traditional claim? The following sections examine phytochemical, toxicological, and pharmacological data to address this inquiry. Rather than viewing these historical accounts as mere empirical anecdotes, modern science provides a critical link to explain them: the host-dependent therapeutic variations documented in ancient texts are directly attributable to the specific metabolic fingerprints transferred from different host trees [12]. While these historical records provide invaluable empirical guidance, they lack the molecular and toxicological evidence required by modern pharmacology. The following section will critically re-evaluate these ancient claims through the lens of contemporary scientific research.

## 4. Modern Research on Sangjisheng: Validating Historical Wisdom and Expanding Quality Dimensions

While ancient texts provide invaluable empirical guidance on the host-dependent quality of Taxilli Herba, they inherently lack the molecular and toxicological evidence required by modern pharmacology. The following sections will bridge this gap by critically re-evaluating these ancient claims through the lens of contemporary phytochemical, toxicological, and biophysical research.

### 4.1. Chemical Variations: Validating the “Superiority of Mulberry Host”

Ancient texts, such as the Compendium of Materia Medica, explicitly revered mulberry as the “authentic host,” asserting that Sangjisheng harvested from mulberry trees possesses superior medicinal efficacy. Modern phytochemical evidence directly validates this historical claim. Modern research has extensively elucidated the multifaceted impact of host plant species on the constituents of Sangjisheng. This influence is primarily manifested in two key aspects: the modulation of the parasite’s inherent metabolic profile and the uptake and accumulation of secondary metabolites from the host. Regarding the inherent components of Sangjisheng, extensive research data indicate significant variations in the total flavonoid content of Sangjisheng samples derived from different host species [17]. Notably, studies have demonstrated that samples hosted by mulberry trees possess significantly higher total flavonoid content compared to those from other hosts [18]. Since flavonoids are recognized as the primary bioactive substances, these quantitative differences are directly correlated with medicinal efficacy [19], providing a critical basis for the quality evaluation and selection of superior germplasm resources.

Furthermore, numerous research findings have unveiled the complexity of the translocation of host components to the parasite. While ancient texts broadly warned against toxic hosts, modern science precisely identifies the specific chemical culprits. Liu et al. [20] identified cardiac glycosides derived from oleander within Sangjisheng parasitizing this host, highlighting the potential safety risks posed by toxic host trees. Conversely, Hu et al. [21] detected 1-deoxynojirimycin in samples parasitizing mulberry trees, while Zhang et al. [22] identified mangiferin in those from mango trees, illustrating the specific transfer of host-specific markers. Notably, Lu et al. [23] revealed that the salicin content in Sangjisheng parasitizing willow trees was 1–2 times higher than that in the host branches themselves, demonstrating a significant bio-enrichment effect. This finding offers new insights into the host–parasite relationship and the potential for biotechnological utilization.

Corroborating these observations, Chai et al. [24] detected cardenolides in *Scurrula parasitica* L. (Loranthaceae) parasitizing oleander, reinforcing the conclusion regarding the transfer of toxic components. Additionally, Zhou et al. [12] employed a widely targeted metabolomics approach to simultaneously analyze Sangjisheng from mulberry, willow, and cinnamon hosts. They discovered that the parasite accumulated characteristic metabolites from each respective host: 1-deoxynojirimycin (DNJ), salicylic acid, and cinnamaldehyde. This comprehensive metabolomic perspective reveals the universality and complexity of component translocation. Collectively, these findings indicate that host species differences not only result in variations in inherent components but also facilitate the uptake of host metabolites. This phenomenon serves as a major determinant of quality divergence in medicinal materials. Crucially, the transmission of toxic components from poisonous hosts—such as cardiac glycosides from oleander—significantly heightens safety risks, suggesting that “host origin” must be a primary criterion in the quality control and safe clinical application of Sangjisheng.

Synthesizing these findings, we can delineate the qualitative similarities and differences in chemical composition across different host varieties, addressing the core mechanism of quality variation:

(1) Qualitative Similarities (The Inherent Baseline)

Regardless of the host species, Sangjisheng maintains a conserved metabolic profile dominated by flavonoids. While the total content may fluctuate significantly depending on the host [17,18], the fundamental classes of bioactive compounds remain consistent, constituting the intrinsic quality baseline of the medicinal material.

(2) Qualitative Differences (The Host Imprint)

Distinct qualitative variations arise primarily from the translocation of host-specific secondary metabolites. Research confirms that Sangjisheng acquires unique chemical markers—such as 1-deoxynojirimycin from mulberry [21], salicin from willow [23], or cinnamaldehyde from cinnamon [12]—which are absent in samples from other hosts. Furthermore, the uptake of toxic components like cardiac glycosides from oleander [20,24] represents a critical qualitative safety difference. Thus, the “host imprint” not only diversifies the chemical spectrum but also dictates the specific efficacy and toxicity profiles of the parasite.

### 4.2. Toxicological Risks: Scientific Basis for Ancient “Taboos”

Historical records, accumulated from centuries of clinical practice, have long established a dual narrative regarding Sangjisheng: while generally regarded as a safe, “superior” tonic, historical records explicitly warned against samples harvested from “miscellaneous” or “poisonous” trees [25]. Modern toxicological research has moved beyond anecdotal warnings to critically validate these ancient taboos through rigorous chemical and biological evidence. The hypothesis that toxic hosts may transfer harmful constituents to the parasite—effectively turning a remedy into a poison—has prompted extensive modern investigation. The first documented instance of toxic component translocation involved *Coriaria napalensis* Wall. During chemical analysis, researchers successfully identified three neurotoxic sesquiterpene lactones—coriamyrtin, tutin, and coriatin—within the parasite [26]. Furthermore, Zhou et al. [27] investigated the toxicity of *Taxillus sutchuenensis* parasitizing various hosts, including mulberry, oleander, lacquer tree, sand pear, plum, poplar, and oak. Their findings revealed that *T. sutchuenensis* growing on toxic hosts (e.g., oleander and lacquer tree) exhibited significant toxicity, causing severe hepatic and renal tissue damage, whereas samples from non-toxic hosts showed negligible toxicity. These studies critically confirm that the “safety” of Sangjisheng is not intrinsic but is entirely conditional upon the host species.

To systematically evaluate these risks and bridge the gap between historical observation and modern quantification, our group previously employed zebrafish (*Danio rerio*) embryos as robust in vivo experimental models to assess the hepatorenal [14], embryonic [28], and cardiac developmental toxicity [15] of Sangjisheng derived from six host trees: *M. alba*, *Salix babylonica*, *Camellia oleifera*, *Nerium indicum*, *Melia azedarach*, and *Toxicodendron trichocarpum*. By integrating endpoints for acute toxicity, development, cardiac function, hepatic injury, and apoptosis from three independent studies, we constructed a 25-point semi-quantitative toxicity scoring system (Table 2). The results were striking and directly aligned with the severity of host toxicity. Sangjisheng sourced from *M. alba* exhibited the lowest cumulative toxicity (5/25). In contrast, samples from *N. indicum*, *M. azedarach*, and *T. trichocarpum* each scored ≥ 20/25, indicating severe embryonic and organ-specific risks (Table 3). Notably, modern evidence also identifies risks that may have been overlooked in ancient records. *S. babylonica* and *C. oleifera* showed similar toxicity profiles with total scores of 13 and 14, respectively. Both exhibited moderate risks across development, cardiac function, and hepatic injury (scoring 3 in each domain), distinguishing them from the highly toxic group but indicating that greater caution is needed compared to *M. alba* (Table 3).

These data underscore that host identity is a critical determinant of Sangjisheng safety, necessitating a re-evaluation of current quality standards. Crucially, the findings suggest that current Chinese Pharmacopoeia screening methods, which focus primarily on cardiac glycosides, may be insufficient to exclude all potentially harmful sources. The “host-like toxicity” observed is not solely attributable to cardiac glycosides; other unknown toxic constituents and mechanisms are likely involved (Table 4). Therefore, to ensure clinical safety, modern quality control must evolve from simple morphological identification to a comprehensive, host-specific toxicological screening protocol.

### 4.3. Pharmacological Properties: Deciphering the Host-Induced “Four Natures”

The traditional concept of “Four Natures” (cold, hot, neutral) is not merely philosophical; however, modern biophysical analysis reveals it as a quantifiable metric of metabolic activity. The classification of “Four Natures” is a fundamental theory in Traditional Chinese Medicine (TCM), reflecting the wisdom of correcting bodily imbalances and restoring harmony [30]. A critical discrepancy exists in current standards: historically, classical texts described Sangjisheng—specifically the variety parasitic on mulberry trees—as having a neutral property. Conversely, the current Chinese Pharmacopoeia generalizes this classification, defining Sangjisheng from all host sources as neutral, despite acknowledging its diverse botanical origins [1].

To resolve this conflict between historical specificity and modern generalization, a pivotal study utilized microcalorimetry to analyze Sangjisheng derived from hosts with distinct natures: warm/hot (e.g., cinnamon trees), cold/cool (e.g., willow trees), and neutral (e.g., mulberry trees) [31]. The results demonstrated a clear correlation: Sangjisheng parasitic on cinnamon trees exhibited warm/hot characteristics, while that from willow trees displayed cold/cool traits. In contrast, the sample from mulberry trees remained neutral. These findings critically challenge the current pharmacopoeial standard, suggesting that the “neutral” designation is an oversimplification that fails to account for host-induced metabolic shifts.

Beyond thermal properties, this host dependency extends directly to therapeutic efficacy, where the host acts as a decisive variable in clinical outcomes. Extensive experimental evidence highlights the impact of host trees on the efficacy of Sangjisheng. For instance, regarding anti-osteoporosis activity, Sangjisheng derived from cinnamon trees was found to be significantly more potent than that from camphor or mulberry trees [32]. In contrast, in studies on hypotensive effects involving six host species (mulberry, willow, chestnut tree, liquidambar tree, *C. oleifera*, and oleander), all but the oleander-derived samples exhibited blood pressure-lowering properties, suggesting a broader range of natural resources for hypertension treatment [33]. Furthermore, in the context of rheumatoid arthritis, Sangjisheng parasitic on mulberry trees demonstrated superior efficacy in ameliorating symptoms in murine models compared to that parasitic on *Liquidambar formosana* [34]. Additionally, research on *Phragmanthera capitata* (a related species) revealed that specimens from *Citrus aurantifolia* hosts possessed the strongest antibacterial activity against *Escherichia coli* and *Staphylococcus aureus*, whereas those from *Cola nitida* were the least effective [35]. Collectively, these data argue against a “one-size-fits-all” application of Sangjisheng. To provide a comprehensive overview of these host-dependent variations, Table 5 summarizes the major host species, their specific chemical markers, pharmacological effects, and toxicity profiles. As indicated in the “Recommended Use Status” column, only *M. alba* is recommended for clinical use due to its optimal balance of efficacy and safety, whereas others pose significant risks or lack sufficient evidence.

### 4.4. Re-Evaluating Toxicity: Limitations and Confounding Factors

Despite the widespread clinical application of Sangjisheng, our analysis reveals significant discrepancies between traditional safety profiles and modern toxicological data. These inconsistencies primarily stem from two critical issues: the limitations of current quality control standards and the ambiguous toxicity of intermediary hosts.

#### 4.4.1. The “Detection Window” Limitation in Toxicity Screening

One major source of conflicting evidence lies in the specificity of current screening methods. As highlighted in our review, the Chinese Pharmacopoeia primarily mandates screening for cardiac glycosides to prevent toxicity from hosts like *N. indicum* [1]. However, our data (Table 3) reveal a critical blind spot: hosts such as *M. azedarach* and *T. trichocarpum* score severely high (≥20/25) on our toxicity scale despite lacking cardiac glycosides.

Recent comprehensive reviews indicate that the toxicological mechanisms of many non-glycoside constituents in Sangjisheng remain poorly understood [8,19]. For instance, *M. azedarach* contains limonoids (e.g., azadirachtin) and *T. trichocarpum* contains urushiols, both of which are potent allergens or neurotoxins not detected by standard cardiac glycoside assays [36]. This suggests that the current safety paradigm is insensitive to non-cardiac glycoside toxins, potentially leading to a false sense of security where “glycoside-negative” samples may still harbor severe hepatotoxic or allergenic risks [14]. Furthermore, even for traditionally safe constituents like lignans, modern in silico predictions suggest potential structural alerts for toxicity that contradict historical safety data [37,38].

#### 4.4.2. The “Grey Zone” of Intermediary Hosts

Another inconsistency involves hosts that are moderately toxic, specifically *S. babylonica* and *C. oleifera*, which scored 13 and 14, respectively, in our assessment (Table 3). While *M. alba* is clearly safe and *N. indicum* is clearly toxic, the clinical implications of these “intermediary” hosts remain ambiguous.

On one hand, these hosts are often cited for their beneficial bioactive compounds; for example, *S. babylonica* (willow) is rich in salicin, which provides well-documented anti-inflammatory benefits [39]. On the other hand, emerging empirical evidence contradicts their absolute safety. Studies using zebrafish models have demonstrated that willow-derived Sangjisheng can induce significant hepatotoxicity and developmental abnormalities at certain concentrations [14]. This contradiction likely arises because the dose–response relationship and the bioaccumulation of host-specific toxins (such as saponins in *C. oleifera*) are poorly defined in the context of parasitic interaction. Consequently, the historical literature may deem them safe based on low-dose usage, while modern high-sensitivity assays flag them as hazardous.

#### 4.4.3. The Overlooked Impact of Environmental Factors

Finally, the variability in toxicity reports may also be attributed to environmental factors that are vastly underexplored. The parasitic nature of Sangjisheng makes it highly susceptible to the host’s soil environment. Research on other medicinal plants suggests that soil composition and heavy metal pollution can significantly alter the metabolic profile and toxicity of the parasite [40]. Furthermore, the role of the endophytic microbiome—which possesses the metabolic capacity to biotransform host compounds into toxic metabolites—is often overlooked in standard phytochemical analyses [41]. Therefore, the “inconsistencies” observed in the literature may not be experimental errors, but rather reflections of complex ecological interactions that current monoculture-based toxicity models fail to capture.

### 4.5. Beyond the Host: A Holistic View of Quality-Determining Factors

The quality of the medicinal herb is primarily determined by its harvesting and post-harvest processing. This encompasses harvesting at the optimal season and considering the plant’s age to ensure sufficient accumulation of bioactive compounds. Furthermore, the methods used for processing and drying are critical and must be scientifically controlled to prevent the degradation of active ingredients, thereby guaranteeing the herb’s stability and safety [42]. While these post-harvest processes provide initial quality control, the herb’s unique properties are further shaped by its ecological context, which extends beyond the host plant alone.

Secondly, the environmental conditions of the origin and associated biological factors form the foundation of the herb’s quality. The unique soil composition, climate patterns, and altitude of specific growing regions provide a distinct ecological background. Additionally, the choice of host plant species, as well as the management of pests, diseases, and other biotic interactions, profoundly influences the herb’s chemical profile and physiological adaptations [43]. These factors interact synergistically, reflecting a holistic paradigm where quality is determined not only by cultivation techniques but also by the complex interplay between the plant, its host, and the surrounding environment [44]. This integrated perspective transcends traditional host-centric analysis, establishing a comprehensive framework for quality evaluation that accounts for the multifaceted influences shaping the medicinal herb’s Dao-di authenticity and inherent variations in efficacy.

## 5. Strategies for Quality Management of Sangjisheng

### 5.1. Elucidation of Molecular Mechanisms

The quality formation of Sangjisheng is intricately linked to its parasitic lifestyle, characterized by a profound molecular exchange with its host. Through the haustorium, a specialized invasive organ, the parasite not only extracts water and nutrients but also acquires complex interactions involving the transfer of secondary metabolites or precursors transported via the host’s vascular tissues [45,46]. The influx, concomitant with the transfer of genetic material [47] (e.g., HGT [38,48], mRNAs [49], sRNAs [50], and proteins [51,52]), reshapes the parasite’s secondary metabolic network. Consequently, this generates a host-dependent metabolic phenotype—often referred to as metabolic plasticity—that ultimately dictates its medicinal quality [53]. As highlighted in a recent comprehensive review, this bidirectional molecular crosstalk is a fundamental feature of parasitic systems, serving as a critical driver for phenotypic plasticity and chemical diversity [54]. While ancient practitioners empirically recognized that host trees dictated medicinal quality, the specific molecular mechanisms underpinning how different hosts modulate the chemical composition of Sangjisheng have only recently begun to be unraveled.

Recent integrated multi-omics studies have revealed a pronounced “host effect” on the biosynthesis of bioactive constituents in Sangjisheng. Metabolomic profiling demonstrated that Sangjisheng parasitizing mulberry (*M. alba*) accumulated significantly higher levels of flavonoids—such as quercitrin, rutin, and hyperoside—compared to those from *Liquidambar formosana* [55]. Transcriptome analysis identified pivotal structural genes in the flavonoid pathway (e.g., FLS, HCT, and 4CL) whose expression patterns paralleled metabolite accumulation, suggesting that hosts regulate biosynthesis by modulating gene expression [56]. Furthermore, proteomic surveys quantified 533 differentially expressed proteins, with key enzymes like PAL, CHI, and F3H exhibiting abundance trends consistent with the chemical variations, thereby substantiating their regulatory roles [55,56]. Collectively, these findings illustrate that the host shapes Sangjisheng quality through multi-level regulatory networks, providing molecular insights for understanding chemotypic variation.

Despite these phenotypic and transcriptomic observations, the precise upstream signaling mechanisms driving these host-dependent chemical variations in Sangjisheng require further exploration. However, insights from diverse parasitic models provide a robust framework for hypothesizing these interactions. First, regarding metabolite transport, the haustorium functions as a selective symplastic bridge. Studies on Cuscuta have confirmed that massive macromolecular exchanges occur between host and parasite, suggesting that variations in host xylem sap composition directly dictate the metabolic flux in the parasite [57]. Second, in terms of gene regulation, host-specific environmental cues act as potent triggers. For instance, host-derived strigolactones are known to trigger developmental changes in Striga via specific receptor pathways, implying that similar host-derived signals may epigenetically or transcriptionally upregulate key biosynthetic genes in Sangjisheng [58]. Third, signaling pathways involving small RNAs are critical. Recent evidence shows that parasitic plants like Cuscuta can transport small RNAs across the haustorial interface to silence host genes and manipulate host defense responses [59]. This bidirectional molecular crosstalk suggests that different host species may exert distinct epigenetic or transcriptional pressures on Sangjisheng, ultimately shaping its specific chemical profile.

While the host effect is dominant, non-host factors—such as soil composition, heavy metals, harvest season, and drying temperature—may indirectly influence Sangjisheng quality by modulating host metabolism. However, these factors are more amenable to standardization compared to the inherent chemical variability imposed by different host species. Future studies could focus on dedicated medicinal gardens coupled with structural equation modeling (if data permits) to systematically quantify the independent contribution of host effects. This will help avoid the oversimplification of a “host-determinism” paradigm and establish a solid theoretical foundation for improving the quality and safety of Sangjisheng medicinal materials.

### 5.2. Improvement of Quality Standards

The current edition of the Chinese Pharmacopoeia permits Sangjisheng to be sourced from a broad range of host plants [1]. While this inclusive definition accommodates diverse regional resources, it poses substantial challenges to quality consistency and clinical safety. As evidenced in previous sections, the chemical profile and therapeutic efficacy of Sangjisheng vary drastically depending on the host species. More critically, the unregulated harvesting of wild Sangjisheng introduces severe safety risks; apart from hosts containing known cardenolide toxins, many unidentified hosts may harbor other unrecognized toxic constituents, rendering the current “one-size-fits-all” standard inadequate for safety assurance.

Therefore, it is imperative to revise and refine the quality standards for Sangjisheng. A crucial step involves transitioning from a generalized botanical definition to a more stringent, host-specific standard. Specifically, prioritizing mulberry (*M. alba*) as the authentic or premium host source should be formally recognized in quality control protocols. Standardizing the host species would significantly mitigate quality fluctuations driven by biological variation, ensuring consistent pharmacological efficacy. Furthermore, limiting the authorized host sources simplifies the traceability of medicinal materials and enhances the reliability of safety inspections. By establishing clear boundaries for host selection, regulatory bodies can effectively exclude toxic variants, thereby aligning traditional usage with modern safety requirements and ultimately elevating the market competitiveness and clinical reliability of Sangjisheng products.

We acknowledge that the proposal to restrict the official botanical origin solely to *M. alba* relies primarily on historical textual research and preclinical evidence (including in vitro assays, in vivo animal models, and metabolomics), rather than high-level clinical randomized controlled trials (RCTs). According to the hierarchy of evidence-based medicine, while RCTs represent the gold standard for efficacy, safety assessments must also weigh historical human use and toxicological data heavily. The consistent documentation in ancient classics, combined with modern in vivo toxicity data revealing host-specific risks, provides a robust rationale for regulatory intervention. To bridge the gap between these preclinical findings and clinical application, future efforts should prioritize well-designed clinical observational studies to validate these host-specific safety profiles.

To translate these findings into practical quality control, the concept of quality markers (Q-markers) [60] must be integrated into the Sangjisheng evaluation framework. Instead of the current “one-size-fits-all” standard, we propose a host-specific Q-marker strategy. Future research should utilize multi-omics (including DNA barcoding for host authentication) and chemometrics to identify differential Q-markers that link preclinical safety signals to clinical quality control. Specifically, markers exclusively enriched in mulberry-derived Sangjisheng (e.g., specific flavonoids) should be correlated with in vivo bioactivity to establish minimum efficacy thresholds. Simultaneously, strict maximum limits (upper thresholds) must be set for known host-derived toxins (e.g., cardiac glycosides) to ensure safety. By establishing a dual-verification system—combining host identification (authenticity) with host-specific Q-marker quantification (quality)—regulatory bodies can precisely grade Sangjisheng and effectively exclude toxic variants, thereby aligning traditional usage with modern safety standards.

Given these complex challenges, relying on single-marker evaluation is inadequate. Therefore, we propose a “host-governed quality paradigm” (Figure 2) to bridge the gap between traditional identification and modern safety requirements. This schematic diagram highlights that a robust quality control system must evolve from simple identification to a closed-loop management system—spanning from host authentication and chemical profiling to toxicity screening and traceability. By visualizing these interconnected steps, we aim to provide a clear roadmap for minimizing safety risks in future research and industrial applications.

While the zebrafish toxicity models provide valuable preclinical insights and serve as a robust screening tool, it is crucial to acknowledge the limitations of extrapolating these findings directly to human clinical safety. Zebrafish embryos do not fully replicate mammalian reproductive physiology or chronic metabolic processes. Therefore, additional studies are warranted before clinical translation. Specifically, comprehensive mammalian reproductive toxicity assays, chronic toxicity evaluations, and rigorous clinical safety studies are still needed. This is particularly critical for vulnerable populations, such as pregnant women, and for the safety assessment of Sangjisheng-derived edible tea products. Future research must prioritize these mammalian and clinical endpoints to fully substantiate the host-specific safety recommendations proposed in this review.

### 5.3. Promotion of Artificial Cultivation

In the realm of Sangjisheng research, our preliminary studies have yielded pivotal insights into its biological characteristics. We identified that Sangjisheng seeds are recalcitrant [61], a finding that laid a crucial foundation for subsequent conservation and utilization strategies. Building on this understanding, we optimized germination protocols and successfully established artificial cultivation techniques for *T. chinensis* [62]. This technological breakthrough paves the way for the standardized cultivation of Sangjisheng on a unified host—specifically *M. alba*—providing essential technical support for the authentic sourcing and sustainable development of this medicinal material.

The successful implementation of artificial cultivation holds profound implications for the industry. Primarily, it alleviates the critical shortage of wild mulberry-hosted Sangjisheng. As market demand surges, the reliance on limited and ecologically fragile wild resources has become a significant bottleneck. Large-scale artificial cultivation ensures a stable and sustainable supply chain to meet pharmaceutical needs. Furthermore, this approach directly addresses the quality heterogeneity and safety risks associated with multi-host wild sources. By standardizing the host to mulberry trees under controlled conditions, we can minimize chemotypic variations and fundamentally eliminate the in vivo toxicological risks derived from unknown or toxic hosts [62].

Ultimately, standardized cultivation not only enhances the intrinsic quality and safety of Sangjisheng but also provides the essential biological foundation for implementing the proposed multi-dimensional quality-control standards (e.g., DNA barcoding and metabolomic fingerprinting). By guaranteeing a consistent supply of high-quality raw materials, it facilitates the revision of pharmacopoeial standards. In alignment with the national “Good Agricultural Practice for Chinese Crude Drugs” (2022 edition) [63], promoting cultivation in geo-authentic regions will ensure that Sangjisheng products meet rigorous quality benchmarks, thereby driving the modernization of traditional Chinese medicine.

### 5.4. Safety Assessment of Edible Resources

Sangjisheng boasts a rich history spanning over 300 years as a traditional tea beverage in the Lingnan region of China [64]. Highly valued by the local populace, it has become an integral part of daily life and folk culture, celebrated for its distinctive flavor and potential health benefits. Internationally, other parasitic plants from the Loranthaceae family, notably European mistletoe [65], are also widely utilized in functional foods, reflecting a growing global consumer interest in natural health products.

The *Lingnan Caiyao Lu* (Records of Medicinal Herbs in Lingnan), a significant historical compendium, documents Sangjisheng collected from eight different host trees (mulberry, pine, liquidambar, tallow, paulownia, sand pear, cypress, and wampee). Crucially, it specifies that only Sangjisheng parasitizing mulberry is suitable for tea consumption [66]. This ancient distinction highlights a profound empirical understanding of host-dependent safety that modern science is now actively validating. Modern studies corroborate this focus on safety and quality. For instance, Liu et al. [67] conducted acute and sub-acute toxicity tests on Sangjisheng tea fermented by *Eurotium cristatum*, confirming its non-toxic nature. Furthermore, research on European mistletoe harvested from five different hosts (poplar, European ash, locust, maple, and black walnut) revealed that specimens from European ash exhibited superior antioxidant properties compared to the others [55]. These findings provide a scientific basis for rigorously selecting raw materials for Sangjisheng tea.

When developing Sangjisheng for edible applications, comprehensive safety and functional evaluations across various host sources are essential. Safety remains the paramount prerequisite; it must be unequivocally established that the material poses no health risks. Concurrently, functional assessments help elucidate its health benefits and unlock its commercial potential. By systematically evaluating Sangjisheng from different hosts, we can identify the highest-quality raw materials and provide scientific guidance for product development. This approach not only meets the rising consumer demand for healthy dietary options but also fosters the diversified and sustainable growth of the Sangjisheng industry.

## 6. Limitations and Expanded Safety Considerations

Beyond the intrinsic toxicity derived from host plants, Sangjisheng faces extrinsic safety risks that warrant rigorous attention. As a parasitic herb, it possesses a unique physiological capacity to hyperaccumulate environmental contaminants. Recent screenings have detected concerning levels of heavy metals (e.g., cadmium, lead) and pesticide residues in wild-harvested batches, reflecting the pollution status of the host’s environment. Furthermore, the endophytic microbiome of Sangjisheng—often overlooked—can produce mycotoxins or interact with the plant’s secondary metabolism to generate unforeseen toxic compounds.

Consequently, future research priorities must expand beyond host identification to include:

(1) Comprehensive environmental monitoring

Establishing strict thresholds for heavy metals and pesticides specific to parasitic herbs.

(2) Microbiome profiling

Utilizing metagenomics to map the endophytic community of Sangjisheng and assessing the safety of microbial metabolites.

(3) Clinical validation

Conducting well-designed clinical trials to validate the safety and efficacy of mulberry-derived Sangjisheng in human populations.

## 7. Conclusions and Future Prospects: Toward a Host-Governed Quality Paradigm for Sangjisheng

Across two millennia of documented use, the quality of Sangjisheng has consistently been viewed through the lens of its host. The present research demonstrates that host identity acts as a biochemical cipher, simultaneously dictating the metabolome, toxicological profile, pharmacological potency, and even the traditional “nature” (cold/neutral/warm) of the parasite. Modern omics technologies now corroborate ancient empirical warnings: non-mulberry hosts can introduce cardiac glycosides, sesquiterpene lactones, or yet-unidentified toxins, whereas mulberry-derived Sangjisheng reproducibly accumulates higher flavonoid levels and exhibits the lowest toxicological score in zebrafish paradigms. These findings strongly suggest that the current Chinese Pharmacopoeia allowance of multiple host sources may pose potential clinical risks and warrants critical re-evaluation.

We therefore propose a host-governed quality paradigm that: (i) restricts official Sangjisheng to mulberry parasites; (ii) establishes a public, open-access repository linking host plant identity to comprehensive, voucher-verified datasets on Sangjisheng; (iii) introduces a Sangjisheng traceability QR Code standard, carrying a tamper-proof label stating the exact host species, harvest location, and batch number linked to a unique digital certificate; and (iv) prioritizes human-plausible toxicology (e.g., hepatic organoids, maternal–fetal transfer models) before any future host expansion.

To achieve this, future research must integrate multi-omics and chemometrics to decode host-dependent metabolic shifts, while leveraging artificial intelligence (AI) to predict toxicity and quality profiles. While implementing this paradigm is highly feasible through standardized cultivation, it faces significant challenges, including the fragmented nature of wild-harvested supply chains, the high cost of building standardized databases, and the lengthy process of updating pharmacopeial standards.

However, it is important to acknowledge that much of the current evidence is derived from historical records, chemical profiling, and preclinical models. While these data provide a compelling scientific rationale, definitive regulatory changes must be preceded by further rigorous human-relevant toxicology, reproductive safety assessments, and large-scale clinical validation. Ultimately, implementing this paradigm will align traditional authenticity with 21st-century safety standards, ensuring that every batch of Sangjisheng delivers the efficacy intended by ancient texts without the hazards introduced by cryptic host chemistry.

## Figures and Tables

**Table 1:** Summary of evidence types supporting the quality and safety evaluation of Sangjisheng.

Evidence Type	Methodology/Approach	Key Contributions to Quality Paradigm	Limitations	Representative Studies
Historical TCM Classics	Literature mining of ancient TCM classics (e.g., Shennong Bencao Jing)	Established the traditional preference for specific hosts (e.g., Mulberry); Defined “Warm/Cold” nature.	Lacks molecular mechanism; Subjective descriptions.	Usage of *Morus alba* as the authentic source since Han Dynasty.
Phytochemical Studies	HPLC, LC-MS isolation and identification	Identified bioactive markers (e.g., flavonoids); Distinguished host-derived metabolites.	Often static; Does not reflect dynamic absorption.	Isolation of quercitrin from *Taxillus chinensis*
Metabolomics	UPLC-Q-TOF-MS, PCA analysis	Revealed global metabolic differences among hosts; Screened differential markers rapidly.	Complex data analysis; Requires validation	Differentiation of Sangjisheng from 5 different hosts based on metabolic profiling.
Animal Studies	Rodent models (SHR rats, inflammation models)	Validated antihypertensive efficacy; Confirmed chronic toxicity profiles.	Species differences; Ethical concerns.	Blood pressure lowering effect in SHR rats treated with mulberry-host extract.
Zebrafish Toxicity	High-throughput screening; Behavioral assays; Heart rate monitoring	Rapid assessment of cardiotoxicity (esp. for Oleander host); Embryotoxicity evaluation.	Simple organism; Metabolic pathways differ from mammals.	Heart failure observed in zebrafish exposed to oleander extracts.
Clinical Evidence	Randomized Controlled Trials (RCTs); Case reports	Confirmed human efficacy and safety; Provided dosage guidelines.	Expensive; Hard to control variables (host origin often unclear).	Clinical trials using standardized Sangjisheng granules for hypertension.

**Table 2:** Scoring criteria for hepatorenal, developmental, and cardiotoxicity assessment in zebrafish embryos (5-point scale).

Endpoint	Source	Scoring Logic
LC_50_ (mg/mL)	[14]	≥5.0 → 1; 3.0–4.99 → 2; 1.0–2.99 → 3; 0.5–0.99 → 4; <0.5 → 5
Developmental Toxicity	[28]	No effect → 1; Slight ↓ → 2; Moderate ↓ → 3; Strong ↓ → 4; Severe ↓ → 5
Cardiotoxicity	[15]	None → 1; Mild → 2; Moderate → 3; Marked → 4; Severe → 5
Hepatotoxicity	[14]	None → 1; Mild → 2; Moderate → 3; Significant → 4; Severe → 5
Apoptosis (heart/liver)	[14]	None → 1; Rare → 2; Focal → 3; Extensive → 4; Massive → 5

**Table 3:** Integrated Toxicity Scores of Taxilli Herba Parasitizing Six Different Host Trees.

Host Plant	LC_50_ Score	Dev. Tox.	Cardio Tox.	Hepato Tox.	Apoptosis	Total/25	Toxicity Level	Characteristic Metabolites	Pharmacological Activities	Clinical Application
*Morus alba*	1	1	1	1	1	5	★☆☆☆☆ Very Low	1-deoxynojirimycin	Potent antihypertensive, anti-osteoporosis, and neuroprotective effects	Recommended
*Salix babylonica*	2	3	3	3	2	13	★★☆☆☆ Moderate	Salicin	Analgesic, antipyretic, and anti-inflammatory (NSAID-like)	Not recommended (chronic & repro tox pending)
*Camellia oleifera*	3	3	3	3	2	14	★★☆☆☆ Moderate	2,4,6-Trihydroxybenzoic acid	Moderate antioxidant and lipid-lowering activities	Not recommended (chronic & repro tox pending)
*Nerium indicum*	4	4	4	4	4	20	★★★★☆ Severe	Enriched in cardenolides (Oleandrin, Adynerin Gentiobioside (ISO), Deacetyloleandrin, Odoroside A, Evomonoside)	Strong cardiotonic (Digitalis-like); narrow therapeutic window	Not recommended (chronic & repro tox pending)
*Melia azedarach*	4	4	4	5	4	21	★★★★☆ Severe	Tetranortriterpenoids (Azadirachtin, Toosendanin)	Anthelmintic and insecticidal (but highly hepatotoxic)	Not recommended (chronic & repro tox pending)
*Toxicodendron trichocarpum*	5	5	4	4	3	21	★★★★☆ Severe	Urushiols and phenolic lipids	None beneficial in this context; strong allergenicity	Not recommended (chronic & repro tox pending)

**Table 4:** Representative non-cardiac glycoside toxic components.

Host Plant	Toxic Constituent	Chemical Type	Detected in Sangjisheng?	Principal Toxicity/Target Organ	Reference
*Coriaria nepalensis*	Tutin	Sesquiterpene lactone	✅	Neurotoxicity: tetanus, convulsions, respiratory arrest	[26]
Same as above	Coriamyrtin	Sesquiterpene lactone	✅	Same as above; mouse LD_50_ 1.2 mg kg^−1^ (i.p.)	[26]
Same as above	Coriatin	Sesquiterpene lactone	✅	Central excitation, clonic convulsions	[26]
*Melia azedarach*	Azadirachtin	Tetracyclic triterpenoid	✅	Hepatotoxicity, embryotoxicity; rat LD_50_ 13.6 mg kg^−1^ (p.o.)	[14,28]
Same as above	Nimbin, Salannin	Triterpene lactone	✅	Hepatic microsomal enzyme inhibition, apoptosis	Same as above
*Toxicodendron trichocarpum*	Urushiol	Alkyl-catechol	✅	Strong sensitizer, hepato-renal tubular necrosis	[12,14]
*Pinus* spp.	Isocupressic acid	Diterpene acid	⚠ (reported in European pine mistletoe)	Abortifacient, uterine smooth-muscle stimulation	[29]

Note: ✅ represents Yes; ⚠ represents warning.

**Table 5:** Comprehensive evaluation and final verdict on the usability of Sangjisheng from different host plants.

Host Species	Chemical Class (Summary)	Key Safety Concern (Risk Factor)	Therapeutic Value	Final Recommendation
*Morus alba*	Flavonoids & DNJ derivatives	None significant	High (BP lowering, Bone health)	Authentic Source (Recommended)
*Salix babylonica*	Phenolic glycosides (Salicin)	Reproductive toxicity potential	Moderate (Analgesic)	Substitute (Use with caution)
*Camellia oleifera*	Triterpenoid saponins	Chronic toxicity signals	Low/Moderate	Not Recommended
*Nerium indicum*	Cardiac glycosides	Severe Cardiotoxicity	Narrow therapeutic window	Toxic Adulterant (Banned)
*Melia azedarach*	Limonoids (Toosendanin)	Hepatotoxicity	Anthelmintic (External use only?)	Toxic Adulterant (Banned)
*Toxicodendron trichocarpum*	Urushiols	Severe Allergenicity	None (in this context)	Toxic Adulterant (Banned)

## Data Availability

New data were generated and analyzed in this study.
